# Cluster Analysis of Healthcare Utilization Patterns in Patients with Comorbid Chronic Obstructive Pulmonary Disease and Atrial Fibrillation

**DOI:** 10.3390/jcm15041444

**Published:** 2026-02-12

**Authors:** Stanislav Kotlyarov, Alexander Lyubavin

**Affiliations:** 1 Department of Nursing, Ryazan State Medical University, 390026 Ryazan, Russia; alexlubavin48@gmail.com; 2 Lipetsk City Hospital №4, 398006 Lipetsk, Russia

**Keywords:** chronic obstructive pulmonary disease, atrial fibrillation, comorbidity, cluster analysis, phenotypes, healthcare utilization patterns, outcomes, mortality

## Abstract

**Background/Objectives**: This study aimed to use cluster analysis of healthcare utilization patterns to identify distinct clinical phenotypes in patients with comorbid chronic obstructive pulmonary disease (COPD) and atrial fibrillation (AF) and to assess their associations with demographic characteristics and clinical outcomes. **Methods**: A retrospective cohort study was conducted using data from 1247 patients with COPD and AF extracted from a regional medical information system (Lipetsk Region, period 2021–2025). The k-means algorithm was used to cluster patients based on the average number of medical encounters per three-character ICD-10 categories. Groups were compared using descriptive and analytical statistical methods with correction for multiple comparisons. **Results**: The k-means algorithm identified three distinct clusters (phenotypes), which differed significantly in demographics, comorbidity structure, and mortality. Cluster 1 (“High-frequency utilization phenotype”, 25.3%): characterized by high utilization for acute respiratory infections, metabolic, and urological diseases; demonstrated the lowest mortality (10.1%). Cluster 2 (“Cerebrovascular Phenotype”, 32.3%): characterized by chronic cerebrovascular pathology and its sequelae (codes I67, I69); had intermediate mortality (20.8%). Cluster 3 (“Low-frequency utilization phenotype”, 42.4%): distinguished by minimal utilization for “outpatient” reasons alongside the highest mortality (31.1%) and a high proportion of deaths from respiratory failure. Analysis within the deceased patient subgroup confirmed the persistence of specific utilization patterns for each phenotype right up until the fatal outcome. **Conclusions**: Cluster analysis of real-world clinical practice data identified three discrete phenotypes of patients with comorbid COPD and AF, which have fundamentally different clinical–behavioral trajectories and prognoses. These findings justify the need for differentiated organizational approaches, particularly the development of proactive strategies for the active detection and engagement in follow-up care of patients with the low-frequency utilization phenotype, which is associated with the worst outcomes.

## 1. Introduction

Chronic obstructive pulmonary disease (COPD) and atrial fibrillation (AF) represent a significant comorbid pair frequently encountered in clinical practice [1]. The prevalence of AF among patients with COPD substantially exceeds that in the general population [2,3]. This comorbidity is not coincidental: both diseases share common risk factors, such as smoking, as well as shared pathophysiological mechanisms, including systemic inflammation, oxidative stress, and hypoxemia, which contribute to remodeling in both lung tissue and the myocardium [4,5].

Patients with comorbid COPD and AF often experience a more severe course of both conditions. These patients are characterized by more frequent and prolonged exacerbations, more pronounced symptoms, lower quality of life, and an increased risk of hospitalizations compared to patients with COPD alone [3,6,7,8]. The progression of COPD often leads to pulmonary hypertension and the development of cor pulmonale, which worsens the clinical course of AF, complicates cardioversion attempts, and more frequently predisposes to a permanent form of AF [9].

Modern approaches to managing patients with COPD increasingly account for the heterogeneity of the disease, aiming to identify clinically significant phenotypes—groups of patients sharing common characteristics that allow for predicting disease course and treatment response [10,11,12,13,14,15]. However, despite the recognized importance of the COPD and AF comorbidity, this patient group remains insufficiently studied from the perspective of internal heterogeneity. Most research treats them as a single cohort, while it is likely that subgroups with distinct clinical profiles, treatment needs, and prognoses exist within it. It is important to note that alongside biological phenotypes, increasing attention is being paid to behavioral patterns, particularly the characteristics of a patient’s interaction with the healthcare system. Identifying “healthcare utilization phenotypes” may be no less important for predicting outcomes and planning care, especially for patients with multiple chronic conditions [16,17,18,19,20,21]. This is due to the fact that both COPD and AF require ongoing monitoring and treatment adjustments. Furthermore, they are often associated with other chronic diseases also requiring treatment control. In this context, patient healthcare utilization patterns may play a significant role in treatment effectiveness and prognosis. It is known that COPD patients’ adherence to treatment varies, many COPD patients do not receive adequate treatment, and non-compliance with the treatment regimen can lead to hospitalizations [22,23,24].

Patterns of healthcare utilization are increasingly recognized as a valuable source of information for understanding the actual course of chronic diseases [25,26,27]. However, in patients with combined pathological conditions, such as COPD and AF, these behavioral patterns remain insufficiently studied. Most often, the subject of research interest is the assessment of one disease on the course of another, including the frequency of seeking medical care. COPD exacerbations, for example, increase the risk of hospitalization for patients with concomitant AF [28]. These data are of great clinical importance, allowing the assessment of clinical associations. However, in addition to these data, patient engagement in healthcare is also important. In this study, we define “healthcare utilization phenotypes” as distinct, clusterable patterns of patient interaction with the healthcare system, characterized by specific frequencies, reasons, and profiles of visits to the doctor that are associated with clinical outcomes. Identifying these phenotypes is clinically interesting because it gives us new insights into the diversity of this complex group of patients, which helps us better plan how to work with them, focusing on those who do not often seek medical care.

In this regard, the aim of this study was to cluster patients with comorbid COPD and AF based on data regarding healthcare utilization for various disease classes (ICD-10) to identify clinically significant phenotypes and subsequently assess their differences in demographic indicators and outcomes.

## 2. Materials and Methods

### 2.1. Study Design and Population

This retrospective cohort study utilized anonymized data from patients with COPD and AF who sought medical care in the Lipetsk Region. The observation period spanned four years, from 1 February 2021 to 31 January 2025. The analysis utilized data obtained from the regional medical information system “Kvazar” (LLC “Medsoft”, St. Petersburg, Russia), which contains information on diagnoses, dates and reasons for seeking medical care, as well as causes of patient death. All medical organizations in the Lipetsk Region are connected to this information system, and data are entered into it by the physicians whom the patients consulted. Physicians undergo training on the correct procedures for data entry into the medical information system, and data accuracy is verified by independent experts as part of treatment quality control.

The study included patients with established diagnoses of COPD (ICD-10 code J44) and atrial fibrillation (ICD-10 code I48). Data from 1247 patients with both COPD and AF were included in the study, among whom 773 (61.99%) were male; the mean age of the patients was 71.82 ± 9.31 years.

Inclusion criteria: Age ≥ 18 years, confirmed diagnoses of AF and COPD according to current clinical guidelines.

Exclusion criteria: Absence of a confirmed diagnosis of AF or COPD.

### 2.2. Data Collection and Variables

The following data were extracted from medical records:

- Demographic indicators: age, sex.
- Mortality data (date of death), cause of death (ICD-10 code).
- Number of medical encounters (dates of encounters), classified by ICD-10 codes from various disease chapters.

For each patient, the average number of encounters per each ICD-10 code over the entire observation period was calculated (in case of patient death, the number of medical encounters during the patient’s lifetime within the observation period was analyzed). The current study analyzed all codes from all ICD-10 classes for which patients sought medical care. The ICD-10 classification was used to better standardize data, given that at the time of the study, this classification was the international standard recommended by the World Health Organization (WHO) and was used for reporting and statistics in healthcare in many countries around the world. In the current study, healthcare utilization was defined as the intensity of recorded healthcare contacts, operationalized as the mean number of coded medical encounters per three-character ICD-10 category during the observation period.

### 2.3. Statistical Analysis

The variable used for clustering was the intensity of healthcare use for specific conditions, measured as the average number of encounters per three-character ICD-10 category (e.g., J44, I48, E11). This approach allowed us to identify groups of patients differing not only in their comorbidity spectrum but also in the nature and frequency of their interaction with the healthcare system. A two-stage approach was used to cluster patients in or-der to improve the validity and interpretability of the results. In the first stage, the optimal number of clusters was determined: a dendrogram analysis was constructed using agglomerative hierarchical clustering (Ward’s method). Visual assessment of the dendro-gram and calculation of the silhouette score clearly indicated that dividing the sample in-to three clusters (k = 3) was optimal. This number also corresponds to the principle of interpretability and clinical applicability, so at the second stage, the k-means algorithm with a given k = 3 was applied to form the final groups. This method was chosen because of its effectiveness in segmenting large sets of continuous data into non-overlapping groups with clear centers, which corresponds to the goal of identifying discrete phenotypes. Be-fore clustering, the data was standardized using the Z-score normalization method. Principal component analysis (PCA) was used to visualize the clustering results.

For categorical variables, differences between groups (including comparisons of three groups) were assessed using the chi-square test. Comparison of continuous data between groups, depending on the circumstances, was performed using the unpaired Student *t*-test, one-way ANOVA, or the Kruskal–Wallis test (for comparing three groups). When comparing three groups, the Bonferroni correction was applied for assessing differences. A *p*-value of <0.05 was considered statistically significant.

Data analysis and statistical processing were performed using the “MedCalc (version 23.4.8)” software by MedCalc Software (https://www.medcalc.org) and the SciPy library for the Python (version 3.11) programming language (https://scipy.org).

### 2.4. Ethical Aspects

The study was conducted in compliance with the ethical norms set forth by the Declaration of Helsinki, using anonymized data, which precludes the possibility of patient identification. The study was approved by the Ethics Committee of the Ryazan State Medical University (protocol No. 4 dated 9 October 2023).

## 3. Results

### 3.1. Clinical and Demographic Characteristics of Patients

The conducted analysis revealed that the patients with COPD and AF included in the study had various comorbid conditions across most ICD-10 chapters for which they sought medical care (Table 1).

### 3.2. Clustering and Cluster Characteristics

The k-means method, applied to the number of medical encounters across different three-character ICD-10 categories, enabled the identification of three clusters of patients with COPD and AF, which significantly differed in their comorbidity profiles and demographic indicators. The distribution across clusters was as follows: Cluster 1—316 (25.3%) patients, Cluster 2—403 (32.3%) patients, Cluster 3—528 (42.4%) patients. The analysis revealed statistically significant differences between clusters in terms of age, sex, and mortality. Patients in Cluster 2 were significantly older than patients in Clusters 1 and 3. The proportion of males progressively increased from Cluster 1 to Cluster 3. The mortality rate in Cluster 3 was significantly higher than that in Cluster 1 (OR 3.99, 95% CI 2.63–6.22) and Cluster 2 (OR 1.71, 95% CI 1.25–2.35) (Table 2).

### 3.3. Healthcare Utilization Profiles by ICD-10 Categories in Clusters

**Figure 1:**
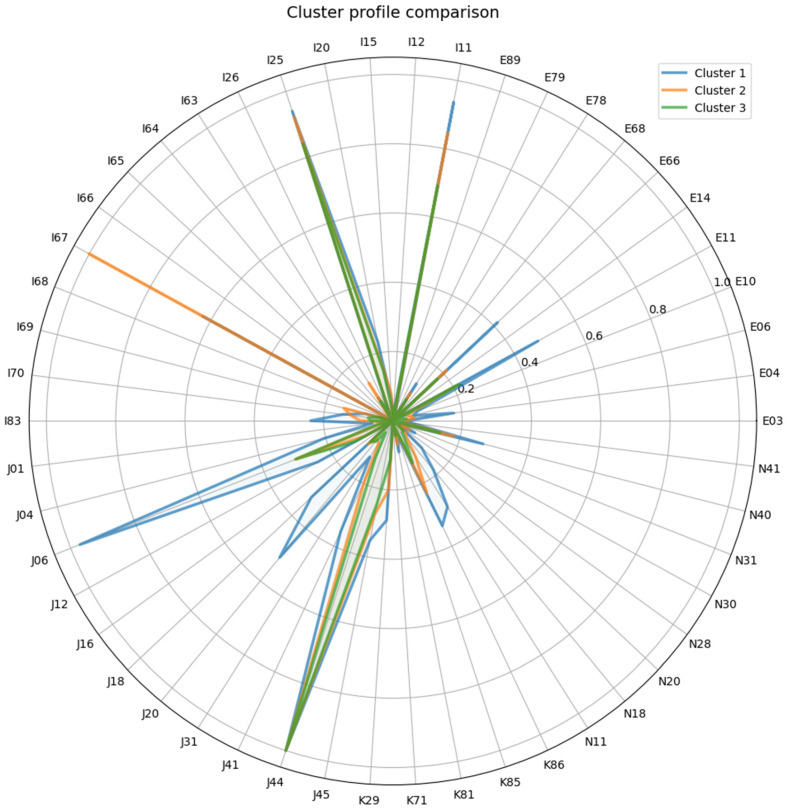
Structure of reasons for medical encounters in the clusters. Comments: The diagram shows the most common ICD-10 disease codes for which patients in each cluster sought medical care. ICD-10 disease codes: I11—Hypertensive heart disease; I12—Hypertensive renal disease; I15—Secondary hypertension; I20—Angina pectoris; I25—Chronic ischemic heart disease; I26—Pulmonary embolism; I63—Cerebral infarction; I64—Stroke, not specified as hemorrhage or infarction; I65—Occlusion and stenosis of precerebral arteries, not resulting in cerebral infarction; I66—Occlusion and stenosis of cerebral arteries, not resulting in cerebral infarction; I67—Other cerebrovascular diseases; I68—Cerebrovascular disorders in diseases classified elsewhere; I69—Sequelae of cerebrovascular disease; I70—Atherosclerosis; I83—Varicose veins of lower extremities; J01—Acute sinusitis; J04—Acute laryngitis and tracheitis; J06—Acute upper respiratory infections of multiple and unspecified sites; J12—Viral pneumonia, not elsewhere classified; J16—Pneumonia due to other infectious organisms, not elsewhere classified; J18—Pneumonia, organism unspecified; J20—Acute bronchitis; J31—Chronic rhinitis, nasopharyngitis and pharyngitis; J41—Simple and mucopurulent chronic bronchitis; J44—Other chronic obstructive pulmonary disease; J45—Asthma; K29—Gastritis and duodenitis; K71—Toxic liver disease; K81—Cholecystitis; K85—Acute pancreatitis; K86—Other diseases of pancreas; N11—Chronic tubulo-interstitial nephritis; N18—Chronic kidney disease; N20—Calculus of kidney and ureter; N28—Other disorders of kidney and ureter, not elsewhere classified; N30—Cystitis; N31—Neuromuscular dysfunction of bladder, not elsewhere classified; N40—Hyperplasia of prostate; N41—Inflammatory diseases of prostate; E03—Other hypothyroidism; E04—Other nontoxic goiter; E06—Thyroiditis; E10—Type 1 diabetes mellitus; E11—Type 2 diabetes mellitus; E14—Unspecified diabetes mellitus; E66—Obesity; E68—Sequelae of hyperalimentation; E78—Disorders of lipoprotein metabolism and other lipidemias; E79—Disorders of purine and pyrimidine metabolism; E89—Postprocedural endocrine and metabolic disorders, not elsewhere classified.

The identified clusters demonstrated differences in the frequency and structure of reasons for seeking medical care (Figure 1).

#### 3.3.1. Diseases of the Respiratory System (J00–J99)

The greatest inter-cluster differences were recorded in the utilization profile for respiratory diseases. Utilization rates for codes related to acute infections were significantly higher in Cluster 1 compared to Clusters 2 and 3. Specifically, the average number of encounters for code J06 (Acute upper respiratory infections of multiple and unspecified sites) in Cluster 1 was 0.97 ± 0.17 versus 0.30 ± 0.46 in Clusters 2 and 3 (*p* < 0.0001). A similar trend was observed for J20 (Acute bronchitis): 0.51 ± 0.50 in Cluster 1 versus 0.07 ± 0.25 and 0.08 ± 0.27 in Clusters 2 and 3, respectively (*p* < 0.0001). Utilization for code J18 (Pneumonia, organism unspecified) was also highest in Cluster 1 (0.32 ± 0.47 versus ~0.10 in other clusters, *p* < 0.0001).

#### 3.3.2. Diseases of the Circulatory System (I00–I99)

The prevalence of arterial hypertension (I10) showed no significant inter-cluster differences. However, utilization for code I11 (Hypertensive heart disease) was higher in Cluster 1 (0.94 ± 0.24) compared to Cluster 3 (0.69 ± 0.46, *p* < 0.0001). In contrast, the incidence of medical care for code I67 (Other cerebrovascular diseases) was highest in cluster 2 (1.0 ± 0.0), compared to clusters 1 and 3 (*p* < 0.0001). The code I69 (Sequelae of cerebrovascular diseases) was also significantly higher in Cluster 2 (0.15 ± 0.35) compared to other clusters (*p* < 0.01).

#### 3.3.3. Endocrine, Nutritional and Metabolic Diseases (E00–E90)

Cluster 1 was characterized by significantly higher utilization for type 2 diabetes mellitus (E11: 0.48 ± 0.50 in Cluster 1 versus ~0.21 in Clusters 2 and 3, *p* < 0.0001) and obesity (E66: 0.41 ± 0.49 in Cluster 1 versus ~0.20 in other clusters, *p* < 0.0001).

#### 3.3.4. Diseases of the Genitourinary System (N00–N99)

Significant differences were also found for this disease chapter. Utilization for such conditions as N11 (Chronic tubulo-interstitial nephritis), N18 (chronic kidney disease), and N40 (Hyperplasia of prostate) was significantly higher in Cluster 1 compared to Clusters 2 and 3 (for all listed codes *p* < 0.005).

Based on the analysis of the utilization structure, summarized cluster profiles were compiled:

Cluster 1: High utilization for acute respiratory infections (J06, J20), pneumonias (J18), as well as chronic metabolic (E11, E66) and urological (N11, N18, N40) diseases.

Cluster 2: High utilization associated with cerebrovascular pathology (I67, I69).

Cluster 3: Characterized by the lowest utilization rates for most diagnoses, except for outcomes, while demonstrating the highest mortality.

### 3.4. Clinical Characterization of Clusters

Cluster analysis revealed three patterns, two of which were defined primarily by the intensity of interaction with the healthcare system (high and low frequency of utilization), and the third by its substantive focus on managing the consequences of a severe cerebrovascular event.

Cluster 1 (n = 316): “High-frequency utilization phenotype” was characterized by a mean age of 71.4 years and a balanced gender distribution (49.4% men). The comorbidity profile was distinguished by pronounced “outpatient” utilization—patients demonstrated the highest number of encounters for acute respiratory infections (J06, J20), community-acquired pneumonias (J12, J18), and chronic bronchitis (J41, J42), indicating constant and active interaction with the healthcare system regarding exacerbations. A distinctive feature of this cluster was the dominance of metabolic diseases with high utilization for type 2 diabetes mellitus (E11) and obesity (E66), suggesting the presence of a COPD with metabolic comorbidities. A significant frequency of utilization for benign prostatic hyperplasia (N40), chronic kidney disease (N18), and urolithiasis (N20 (Calculus of kidney and ureter)) was also noted. The key characteristic of this cluster was the high frequency of healthcare utilization among patients who had a wide spectrum of chronic but controlled conditions. Despite frequent exacerbations, this phenotype is associated with the most favorable prognosis, as evidenced by the lowest mortality rate (10.1%) among all identified clusters. These findings allow us to consider patients in this cluster as a group with high frequency of healthcare utilization, where regular medical follow-up contributes to timely therapy adjustment and prevention of life-threatening complications.

Cluster 2 (n = 403): “Cerebrovascular Phenotype” included the oldest patients (mean age 73.15 years) with a predominance of men (63.0%). The comorbidity profile was characterized by the dominance of neurological deficit—code I67 (other cerebrovascular diseases) was recorded in virtually all patients, and high utilization for sequelae of cerebrovascular diseases (I69) was noted. These features suggest a history of severe neurological events, likely accompanied by cognitive and motor impairments. The cluster exhibited moderate “respiratory” utilization—utilization rates for acute infections and pneumonias were significantly lower than those in Cluster 1, which may be explained by limited patient mobility and a shift in the focus of medical supervision toward neurological pathology. Low utilization for “outpatient” diagnoses was noted—rates for diabetes, obesity, and urological pathology were comparable to or lower than those in Cluster 3. The key characteristic of the cluster was the dominance of post-stroke condition as the leading clinical problem. These data indicate that patients with this phenotype require long-term care, and their medical contacts are largely driven by neurological deficit. The prognosis for patients in Cluster 2 was intermediate, with a mortality rate of 20.8%, reflecting the chronic, disabling nature of the disease course while retaining a significant risk of fatal outcome.

Cluster 3 (n = 528): “Low-frequency utilization phenotype” was the most numerous and included patients with the youngest mean age (71.06 years) and a predominance of men (68.8%). The comorbidity profile showed minimal treatment for “outpatient” diagnoses—treatment rates for acute respiratory infections (J06, J20), chronic bronchitis (J41), and pneumonia (J18) were the lowest among all clusters. A characteristic feature was also low utilization for controlled chronic diseases—diabetes (E11) and obesity (E66)—which may indicate insufficient medical activity or limited interaction of patients with the healthcare system.

The key characteristic of this cluster was its association with the worst prognosis, confirmed by the highest mortality rate (31.1%). The combination of low utilization rates for most diagnoses with high mortality suggests that patients in this group primarily seek medical care at late, decompensated stages of the disease, often due to the development of life-threatening conditions and severe complications. The significant predominance of men in the cluster aligns with known epidemiological data about men’s lower adherence to preventive examinations and early medical help-seeking. The identified features highlight the need to develop specific approaches for the active detection and management of patients with this phenotype.

The distribution pattern of the preventive examination indicator (ICD-10 code Z01 (Other special examinations and investigations of persons without complaint or reported diagnosis)) across clusters corresponded to their clinical–behavioral profiles. The highest proportion of patients who underwent a preventive examination was observed in Cluster 1 (92.7%), reflecting their high frequency of healthcare utilization (women—150 (93.75%), men—143 (91.66%)). In Cluster 2, 81.1% of patients underwent preventive examination (women—120 (80.53%), men—208 (81.88%)), which is likely related to the need for constant medical supervision due to neurological deficit. Despite the overall high coverage of preventive examinations in the population, Cluster 3 had the lowest proportion −75.37% (women 127 (76.96%), men—271 (74.65%)) (*p* < 0.0001 when comparing proportions between clusters). This means that approximately one in four patients in the group with the worst prognosis was not engaged in the system of preventive follow-up, which further stratifies the risk within this phenotype.

### 3.5. Analysis of Cause of Death Structure in Clusters

A comparative analysis of the cause of death structure in the three identified clusters reveals substantial differences corresponding to their clinical characteristics (Table 3).

Analysis of the mortality structure revealed substantial inter-cluster differences, objectively confirming the clinical relevance of the identified phenotypes. The observed differences fully correspond to the clinical profiles of the clusters. The analysis of mortality structure, expressed as the proportion of patients in a cluster who died from a specific cause, objectively confirms the clinical relevance of the identified phenotypes.

In Cluster 1 (High-frequency utilization phenotype), the leading cause of death in terms of absolute burden was heart failure (I50), accounting for 5.06% of all patients in this group. No deaths from respiratory failure (J96) were recorded, and the proportion of deaths from cerebrovascular pathology was minimal. This possibly indicates more successful secondary stroke prevention under conditions of active medical supervision.

Cluster 2 (cerebrovascular phenotype) was characterized by a significant and specific contribution of cerebrovascular pathology, accounting for 2.72% of patient deaths in this group—a figure significantly exceeding that in Cluster 1 (*p* < 0.001). This logically reflects the main clinical focus of this group. The burden of deaths from heart failure (7.44%) and cor pulmonale (3.47%) was also substantial.

The most concerning picture was observed in Cluster 3 (Low-frequency utilization phenotype). This group demonstrated the highest absolute burden for key causes of death. Heart failure accounted for 10.22% of cluster deaths, respiratory failure for 3.97%, and cerebrovascular causes for 3.97%. All these figures were statistically significantly higher than the corresponding values in Cluster 1. Of particular clinical importance is the high mortality rate from respiratory failure, which distinguishes this cluster from the others and indicates an uncontrolled course of the underlying pulmonary disease. Also noteworthy is the high proportion of non-specific causes of death (R-series)—2.84%—which may serve as a marker of late diagnosis and insufficient lifetime examination of patients with low medical engagement.

Thus, patterns of healthcare utilization not only reflect the characteristics of a patient’s interaction with the healthcare system but are also directly associated with qualitatively different and quantifiable mortality profiles. The greatest absolute burden of key fatal complications is observed in the group with the lowest healthcare utilization, necessitating the development of differentiated preventive strategies for each identified phenotype.

### 3.6. Analysis of Healthcare Utilization Patterns in the Subgroup of Deceased Patients

For an in-depth assessment of the clinical relevance of the identified phenotypes, an analysis of the utilization structure was conducted in the subgroup of patients who died during the observation period (n = 280). This analysis allowed us to evaluate the extent to which the healthcare utilization patterns characteristic of each cluster overall were preserved in the group with the worst outcome, and to identify specific features preceding the fatal outcome.

The utilization profiles of deceased patients differed statistically significantly between clusters and fully corresponded to the general characteristics of the identified phenotypes, acting as their amplified reflection (Table 4).

In Cluster 1 (n = 32), deceased patients demonstrated the highest frequency of healthcare utilization. They sought care for type 2 diabetes mellitus (E11: 0.53 ± 0.51 vs. 0.17 ± 0.37 and 0.20 ± 0.40 in clusters 2 and 3, respectively, *p* < 0.0001), dyslipidemia (E78: 0.12 ± 0.34 vs. 0.05 ± 0.21 and 0.02 ± 0.13, *p* < 0.01), and obesity (E66: 0.19 ± 0.40 vs. 0.08 ± 0.28 and 0.05 ± 0.22, *p* < 0.05) significantly more often than deceased patients from other clusters, confirming the dominance of the metabolic component in the comorbidity structure of this phenotype. They also had the highest utilization rates for chronic kidney disease (N18: 0.31 ± 0.47 vs. ≤0.03 in other clusters, *p* < 0.0001) and urolithiasis (N20: 0.22 ± 0.42 vs. ≤0.04, *p* < 0.0001). The intensity of encounters related to preventive observation (codes Z00-Z13) was also highest in this group. These data indicate that fatal outcomes in this phenotype occurred against a background of a thoroughly documented and actively monitored spectrum of chronic diseases.

In Cluster 2 (n = 84), the utilization profile of the deceased was unequivocally determined by cerebrovascular pathology. Virtually all patients had encounters with the code I67 (“Other cerebrovascular diseases”) (1.0 ± 0.0), which significantly distinguished them from both Cluster 1 (0.47 ± 0.51, *p* < 0.0001) and Cluster 3 (0.0 ± 0.0, *p* < 0.0001). Simultaneously, this group showed increased utilization for venous diseases (I80 (Phlebitis and thrombophlebitis), I83 (Varicose veins of lower extremities)) compared to Cluster 3. This profile confirms that the medical interaction of patients with this phenotype, right up to the fatal outcome, was focused on managing the consequences of severe neurological deficit and associated complications.

In Cluster 3 (n = 164), deceased patients were characterized by the most deficient utilization profile, which was a direct continuation of the general characteristic of the “Low-frequency utilization phenotype”. Despite all patients having a diagnosis of chronic ischemic heart disease (I25), the average intensity of encounters for this reason was significantly lower than that in clusters 1 and 2 (0.76 ± 0.43 vs. 1.0 ± 0.0 and 0.90 ± 0.30, *p* < 0.01). A similar trend was observed for hypertensive heart disease (I11) and diabetes mellitus (E11). At the same time, utilization indicators for sequelae of cerebrovascular diseases (I69) and respiratory failure (J96) in this cluster were comparable to or higher than those in others. This pattern—minimal utilization for “background” chronic diagnoses in the presence of encounters for severe complications—supports the hypothesis that patients with this phenotype interact with the healthcare system primarily at the stage of decompensation and development of life-threatening conditions.

Thus, the analysis of the deceased patient subgroup objectively confirmed that the phenotypes identified based on healthcare utilization data represent stable clinical–behavioral trajectories that retain their specificity right up until the fatal outcome. This underscores the high prognostic value of the cluster model and the necessity of developing differentiated preventive strategies aimed at altering the pattern of interaction with the healthcare system, especially for patients with the low-frequency utilization phenotype.

## 4. Discussion

Using data from real-world clinical practice, we identified three healthcare utilization phenotypes in patients with COPD and AF. These phenotypes, based on patterns of care utilization, have direct prognostic value and indicate the need for differentiated organizational approaches. The key result is not simply stratification by severity, but the identification of qualitatively different clinical trajectories.

Cluster 1 (High-frequency utilization phenotype) is characterized by a combination of high medical care utilization (often for acute infections) and the most favorable prognosis. It can be assumed that this reflects not the severity of the condition, but high healthcare utilization and, probably, better access to medical care. The frequent healthcare utilization for COPD exacerbations and respiratory infections observed in this phenotype may facilitate more thorough diagnostic evaluation and systematic registration of comorbid conditions, such as diabetes mellitus and obesity. The low mortality rate in this group suggests that active outpatient monitoring and timely treatment of exacerbations are an effective management strategy even with a significant comorbidity burden.

Cluster 2 (“Cerebrovascular phenotype”) is determined by the presence of cerebrovascular diseases (codes I67, I69). This is consistent with data showing that AF is a leading risk factor for ischemic stroke, and the presence of COPD further increases this risk and worsens the neurological prognosis. The high average age of patients in this cluster correlates with general population data on the accumulation of risk for cerebrovascular events with age. The intermediate mortality rate probably reflects the chronic, disabling nature of the disease, where the cause of death is not so much acute cardiopulmonary events as long-term complications of neurological deficit and concomitant infections.

Cluster 3 (“Low-frequency utilization phenotype”) presents the greatest clinical and organizational challenge. The current study does not allow us to establish a causal relationship, but the patterns identified, such as minimal healthcare utilization for conditions managed primarily on an “outpatient” basis, low registration of chronic diseases, and high mortality, may support the hypothesis of systemic inaccessibility or low adherence to treatment in this group of patients. At the same time, it cannot be ruled out that patients may have had certain characteristics of their primary and concomitant diseases, the severity of which affected their ability to seek medical care.

The results obtained are consistent with current understanding of the heterogeneity of COPD and the existence of different clinical phenotypes of the disease [29,30]. In particular, the phenotype associated with frequent exacerbations is well described in the literature [31,32]. However, the current study adds important data: the high-frequency utilization phenotype itself is not necessarily prognostically unfavorable if it is accompanied by active medical supervision. The identification of the “cerebrovascular” phenotype highlights the critical importance of stroke prevention in patients with COPD and AF [33,34], which requires strict control of anticoagulant therapy. The low-frequency utilization phenotype essentially identifies a “blind spot” in the healthcare system—a group of patients who are not covered by effective medical supervision, which leads to the worst outcomes.

The identified phenotypes have direct practical significance for risk stratification and personalization of management tactics: for Cluster 1, the priority is to optimize the treatment of exacerbations, respiratory support, and control of metabolic disorders. For Cluster 2, a multidisciplinary approach is needed with an emphasis on neurological rehabilitation, secondary stroke prevention, and care to prevent complications. Cluster 3 requires proactive organizational measures: active identification, programs to increase adherence to treatment, improved access to medical care, and education for patients in risk groups.

The data obtained in the current study are consistent with previously published studies highlighting the problem of insufficient coverage and low utilization of planned medical care by patients with chronic respiratory diseases. There is a significant gap between the objective presence of the disease and its active clinical management. For example, it has been previously demonstrated that only 27.2% of patients with a confirmed diagnosis of COPD sought related care within three years, indicating a systematic underestimation and insufficient monitoring of this disease in the population [35]. Similarly, in another study, only 18.1% of patients with mild and 33.9% with moderate/severe obstruction reported outpatient visits for COPD [36]. A cluster analysis conducted in a previous study identified three different clinical phenotypes of patients in the pre-diagnostic period: “Paucisymptomatic–Preserved,” “Frequent Attender/High-Risk,” and “Silent Decliner.” Patients with asymptomatic deterioration had severe airway obstruction despite moderate symptoms and relatively infrequent medical care seeking prior to diagnosis [37]. In addition, it has previously been shown that in the group of elderly patients with early mortality, there was a moderate or low level of seeking medical care at the initial stage [38]. The authors suggested that some of the deaths may have been related to sudden events (e.g., accidents) or insufficient use of available medical services, which ultimately led to an unfavorable outcome.

Data from previous studies confirm that the nature of a patient’s interaction with the healthcare system is influenced by a complex set of factors that go beyond the severity of the underlying disease. Social and behavioral determinants deserve special attention. Lower socioeconomic status was an independent predictor of COPD-related visits [36], and lack of time creates a critical barrier to regular monitoring even when medical care is affordable, as demonstrated in the case of asthma [39]. The predominance of men in our Cluster 3 is consistent with data on lower adherence to treatment among men in the Russian population [40]. Thus, low-frequency utilization phenotype we identified reflects not only medical but also socioeconomic vulnerability, requiring proactive organizational measures.

Finally, the data presented emphasize the importance not only of the frequency but also of the nature of medical care seeking. It has been previously shown that patients with newly diagnosed or poorly controlled COPD are more likely to use emergency care [41], and the degree of obstruction directly correlates with the risk of calling an ambulance and hospitalization, but not with the frequency of scheduled visits to the doctor [42]. Lower socioeconomic status was significantly associated with a higher frequency of visits to emergency departments for COPD exacerbations [43]. Age, number of comorbidities, hypertension, heart failure, diabetes, syncope, COPD, and chronic kidney disease—all of these factors were significantly associated with an increased risk of emergency department visits, and this association remained even after adjusting for age and gender [44]. In a previous multivariate analysis, frequent visits were identified as an independent factor preventing COPD exacerbations requiring hospitalization in a ward, emergency department, or intensive care unit. In addition, frequent outpatient visits reduce the risk of COPD exacerbations by 45–60% [45].

Patients with AF are also clinically heterogeneous [46]. Previously, it was shown that among patients with heart failure, those with AF had more outpatient visits, more emergency department visits, and more hospitalizations than those without AF [47]. At the same time, rural residents with AF had fewer outpatient visits but more emergency department visits than urban residents [48]. Despite a slight increase in mortality, patients with heart failure who had AF sought medical care significantly more often and spent more on it than those without AF [47]. It was found that as patients age, more specialists may be required to be involved in their treatment. Among patients aged ≥75 years, 20% of patients with AF consulted ≥5 specialists, compared with 5% of patients without cardiovascular disease. Multimorbidity was closely associated with an increased risk of hospitalization (≥4 comorbidities: OR 10.6, 95% CI: 8.4–12.1) and an increased risk of emergency department visits (≥4 comorbidities: OR 6.7, 95% CI: 5.7–7.9) [44]. It has also been shown that among patients with existing AF, acute exacerbations of COPD were associated with a higher risk of visiting the emergency department or being hospitalized for AF within the first 90 days after acute exacerbations of COPD [49].

Thus, our results, obtained from real-world data, integrate and extend existing observations. We not only confirm that low-frequency utilization is a marker of high risk, but also propose a tool for its objective stratification based on routine utilization data, paving the way for the development of targeted interventions for the most vulnerable patient groups. A strength of the study is the use of real-world utilization data, which allowed us to identify not only medical but also behavioral patterns. The large cohort size and statistical significance of the differences lend the results a high degree of reliability. It is important to note that the frequency of healthcare utilization of comorbid patients with COPD and AF in the current context is rarely the subject of research and is still largely unknown at this time. In this regard, the current study provides new data that may be the subject of future detailed studies aimed at improving the quality of medical care.

Despite the novelty and relevance of the data obtained, the current study has several limitations. First, it is a retrospective design, which does not allow establishing causal relationships, making it impossible to determine the reasons for the rare use of medical care. Second, we analyzed data on coded encounters rather than complete medical histories, so some diagnoses, particularly in the low-utilization group, may have been missed. Third, the lack of data on received therapy (e.g., anticoagulants, inhaled corticosteroids), smoking status, lung function parameters (forced expiratory volume in 1 s (FEV1)), and socioeconomic status, which could have deepened the interpretation of the clusters. Furthermore, the limitations of the study design do not allow for the assessment of different types of medical care (hospitalization, outpatient care, emergency care, etc.) and the type of medical organization. It should also be noted that since the main objective of the study was to identify patterns of seeking care rather than to establish independent predictors of mortality, no multivariate analysis was performed in the current study to assess the independent contribution of the identified phenotypes to mortality risk. It should also be noted that the patterns obtained may reflect national characteristics of the healthcare system, which may vary from country to country, which is a promising topic for further research.

## 5. Conclusions

Thus, the current study provides new evidence that healthcare utilization is an effective tool for stratifying patients with COPD and AF. Three distinct clinical healthcare utilization phenotypes with fundamentally different risk profiles were identified. It was found that the “High-frequency utilization phenotype” has a favorable prognosis despite frequent medical care utilization, which emphasizes the protective role of regular medical monitoring. The most vulnerable group is patients with Low-frequency utilization phenotype, who have the worst outcomes. These results justify a shift from a uniform strategy for managing comorbid patients to a differentiated one. The results of the study emphasize the need to develop targeted active intervention programs aimed at this high-risk group. These programs may include the development of measures to identify patients with low-frequency utilization patterns, their coverage, and involvement in medical care programs. Such interventions could also include patient navigation services and individualized education aimed at overcoming barriers to healthcare. Further research should focus on identifying the factors that determine low-frequency healthcare utilization and testing the effectiveness of personalized engagement strategies to improve treatment outcomes in this high-risk group.

## Figures and Tables

**Table 1:** Clinical and demographic characteristics of patients.

Characteristic	Presence of Disease (n = 1247)
Demographic Data	
Age	71.82 ± 9.31 years
Male sex	773 (61.99%)
Clinical Condition	
Cardiovascular Diseases	
Chronic ischemic heart disease	1113 (89.25%)
Arterial hypertension	1002 (80.35%)
Cerebrovascular disease	601 (48.2%)
Angina pectoris	215 (17.24%)
Chronic heart failure	209 (16.76%)
Ischemic stroke	112 (8.98%)
Dilated cardiomyopathy	49 (3.93%)
Pulmonary embolism	25 (2.0%)
Primary MI	19 (1.52%)
Aortic stenosis	13 (1.04%)
Peripheral artery disease	13 (1.04%)
Recurrent MI	8 (0.64%)
Intracerebral hemorrhage	4 (0.32%)
Endocrine Diseases	
Type 2 DM	350 (28.07%)
Obesity	310 (24.86%)
Hypothyroidism	103 (8.26%)
Type 1 DM	53 (4.25%)
Respiratory Diseases	
Acute respiratory viral infection	588 (47.15%)
Bacterial pneumonia	188 (15.08%)
Viral pneumonia	180 (14.43%)
Lung cancer	58 (4.65%)
Other Diseases	
Anemia	65 (5.21%)

**Table 2:** Key demographic indicators and outcomes in the identified clusters.

Parameter	Cluster 1 (n = 316)	Cluster 2 (n = 403)	Cluster 3 (n = 528)	*p*-Value (1 vs. 2)	*p*-Value (1 vs. 3)	*p*-Value (2 vs. 3)
Age, years (M ± SD)	71.4 ± 8.96	73.15 ± 8.72	71.06 ± 9.84	0.0083 ^1^	0.6217	0.0008 ^1^
Male, n (%)	156 (49.4%)	254 (63.0%)	363 (68.8%)	0.0003 ^1^	<0.0001 ^1^	0.0784
Mortality, n (%)	32 (10.1%)	84 (20.8%)	164 (31.1%)	0.0002 ^1^	<0.0001 ^1^	0.0006 ^1^

^1^ *p* < 0.0167—statistically significant difference after applying the Bonferroni correction for three pairwise comparisons (adjusted significance level α = 0.0167).

**Table 3:** Comparative Characteristics of Mortality Causes in Clusters.

Cause of Death	High-Frequency Utilization Phenotype (n = 316)	Cerebrovascular Phenotype (n = 403)	Low-Frequency Utilization Phenotype (n = 528)	Significance of Differences
	1	2	3	
Number of deaths over 4 years	32 (10.1%)	84 (20.8%)	164 (31.1%)	*p* ^1,3^ < 0.001
Sex (M/F) (from the number of deaths)	24/8	62/22	120/44	
Age at death	71.4 ± 10.8 yrs	74.9 ± 9.9 yrs	73.2 ± 9.7 yrs	
Heart failure (I50)	16 (5.06%)	30 (7.44%)	54 (10.22%)	*p* ^1,3^ < 0.001
Cor pulmonale/ Pulmonary heart disease (I27)	5 (1.58%)	14 (3.47%)	28 (5.30%)	
Respiratory failure (J96)	0	6 (1.48%)	21 (3.97%)	*p* ^1,3^ < 0.001
Pulmonary embolism (I26)	3 (0.94%)	3 (0.74%)	9 (1.70%)	
Cerebrovascular pathology (G93.6, F01, I67)	0	11 (2.72%)	21 (3.97%)	*p* < 0.001
Oncological diseases (C80.9)	3 (0.94%)	5 (1.24%)	6 (1.13%)	
Renal failure (N17-N18)	1 (0.32%)	2 (0.49%)	0	
Non-specific causes (R-series)	3 (0.94%)	8 (1.98%)	15 (2.84%)	

Note: Data are presented as n (% of the total number of patients in the cluster).

**Table 4:** Intensity of medical encounters (mean number of encounters per patient) by key ICD-10 chapters among deceased patients in the identified clusters.

ICD-10 Code	Description	Cluster 1 (n = 32)	Cluster 2 (n = 84)	Cluster 3 (n = 164)	*p* (1 vs. 3)	*p* (2 vs. 3)
E11	Type 2 diabetes mellitus	0.53 ± 0.51	0.17 ± 0.37	0.20 ± 0.40	<0.0001	0.587
I11	Hypertensive heart disease	0.97 ± 0.18	0.71 ± 0.45	0.55 ± 0.50	<0.0001	0.015
I25	Chronic ischemic heart disease	1.0 ± 0.0	0.90 ± 0.30	0.76 ± 0.43	0.002	0.007
I67	Other cerebrovascular diseases	0.47 ± 0.51	1.0 ± 0.0	0.0 ± 0.0	<0.0001	<0.0001
J20	Acute bronchitis	0.56 ± 0.50	0.11 ± 0.31	0.03 ± 0.17	<0.0001	0.013
N18	Chronic kidney disease	0.31 ± 0.47	0.02 ± 0.15	0.03 ± 0.17	<0.0001	0.765
Z01	Medical examination/Health check-up	0.88 ± 0.34	0.61 ± 0.49	0.60 ± 0.49	0.003	0.885

Note: Presented *p*-values are for key pairwise comparisons after Bonferroni correction.

## Data Availability

The raw data supporting the conclusions of this article will be made available by the authors on request.
